# Safety of cardiopulmonary exercise testing in pulmonary hypertension: insights from a UK tertiary referral centre

**DOI:** 10.1183/13993003.02207-2025

**Published:** 2026-03-19

**Authors:** Andrea Baccelli, Gulammehdi Haji, Hannah Tighe, Luke S. Howard

**Affiliations:** 1 National Pulmonary Hypertension Service, Hammersmith Hospital, Imperial College Healthcare NHS Trust, London, UK; 2 National Heart and Lung Institute, Imperial College London, London, UK; 3 Respiratory Physiology, Imperial College Healthcare NHS Trust, London, UK

## Abstract

Cardiopulmonary exercise testing (CPET) remains the gold standard for evaluating exercise capacity, offering a comprehensive assessment of the integrative metabolic, cardiovascular and ventilatory responses to exertion [1, 2].

## Shareable abstract

**Incremental, symptom-limited CPET is safe in patients with suspected or confirmed pulmonary hypertension. In over 3800 tests, no deaths or syncopal events occurred, with an adverse event rate of only 0.05%, supporting wider use under proper supervision.** https://bit.ly/4qdZf6x

*To the Editor:*

Cardiopulmonary exercise testing (CPET) remains the gold standard for evaluating exercise capacity, offering a comprehensive assessment of the integrative metabolic, cardiovascular and ventilatory responses to exertion [1, 2].

Despite being widely recognised as a safe investigation, CPET continues to be underused in pulmonary hypertension (PH), largely due to limited availability of experienced laboratories and concerns regarding potential adverse events in this high-risk population with restricted stroke volume augmentation during exercise [3]. However, its diagnostic and prognostic value in pulmonary vascular disease is now well-established [4–10].

Real-world evidence on the safety of CPET in the diagnostic work-up and longitudinal assessment of PH remains scarce. We therefore aimed to evaluate the safety profile of incremental, symptom-limited CPET in a large cohort of patients with suspected or confirmed PH.

We retrospectively reviewed all CPETs performed at Hammersmith Hospital, Imperial College Healthcare NHS Trust, between January 2015 and August 2025 through our electronic records database.

Symptom-limited, incremental CPETs were performed using a standard metabolic cart (CPX; Vyaire Medical, Basingstoke, UK; and Jaeger Medical, Trophy Club, TX, USA) in the upright position on an electromagnetically braked cycle ergometer (Ergoline GmbH, Bitz, Germany), according to the American Thoracic Society (ATS) guidelines and latest recommendations [1, 2]. Reference values for peak oxygen uptake (*V*′_O_2__) were derived using the Wasserman–Hansen equations [11, 12].

At our centre, CPET was contraindicated in patients with recent syncope, inability to cycle, or if needing supplemental oxygen at rest. Tests were performed by physiologists certified in advanced life support, with medical supervision available on site.

Across the 10-year period, we identified 3867 CPETs in 2682 unique subjects. Baseline characteristics of the patient population are summarised in the table 1. Mean±sd age was 56.7±16.4 years (33% ≥65 years; 5% ≥80 years), and 55% were female. The largest majority of patients were diagnosed with World Health Organization group 1 PH (33%). Functional capacity was broadly impaired, with a mean peak *V*′_O_2__ of 17.7±7.3 mL·kg^−1^·min^−1^ (60.4±19.5% predicted), mean minute ventilation/carbon dioxide production (*V*′_E_/*V*′_CO_2__) slope of 40.1±13.6, and peak respiratory exchange ratio of 1.16±0.12, indicating overall good effort. The mean 6-min walk distance was 324 m. Approximately 15% of patients achieved a peak *V*′_O_2__ <11 mL·kg^−1^·min^−1^, consistent with high risk and severe functional limitation. Adopting a recently proposed CPET score to stratify risk, integrating peak *V*′_O_2__, O_2_ pulse and *V*′_E_/*V*′_CO_2__ slope, 387 (10%) tests were performed in high-risk subjects, and just over a third of examinations were performed in an intermediate–high- to high-risk group of patients, as shown in table 1 [5]. 2514 out of 3867 (65%) tests achieved criteria for maximality [2].

**TABLE 1:** Patient characteristics

**Age, years**	56.7±16.4
**Female**	2127 (55%)
**WHO-FC**	
N/A	574 (15%)
I	306 (8%)
II	680 (18%)
III	2139 (55%)
IV	168 (4%)
**Defined PH diagnosis**	
Group 1	1272 (33%)
Group 2	248 (6%)
Group 3	145 (4%)
Group 4	920 (24%)
Group 5	52 (1%)
Suspected PH	1231 (32%)
**6MWD, m**	324±145
**Cardiopulmonary exercise testing**	
Peak *V*′_O_2__, mL·min^−1^·kg^−1^	17.7±7.3
Peak *V*′_O_2__, % predicted	60.4±19.5
*V*′_E_/*V*′_CO_2__ slope	40.1±13.6
Peak O_2_ pulse, mL·beat^−1^	9±3
Peak O_2_ pulse, % predicted	66±12
RER	1.16±0.12
Peak HR, beats·min^−1^	145±26
Peak HR, % predicted	88±14
*P*_etCO_2__, kPa	3.21±0.63
**CPET score risk**	
Low	1817 (47%)
Intermediate–low	696 (18%)
Intermediate–high	967 (25%)
High	387 (10%)
**Right heart catheterisation (n=1977; 74% of patients)**	
Mean RAP, mmHg	9±4
Mean PAP, mmHg	45±15
Mean PAWP, mmHg	11±5
Cardiac output, L·min^−1^	4.6±2
Cardiac index, L·min^−1^·m^−2^	2.4±1
PVR, Wood unit	8.4 (5.2–12.8)

Data are reported as mean±sd, n (%) or median (interquartile range). WHO-FC: World Health Organization functional class; N/A: not available; PH: pulmonary hypertension; 6MWD: 6-min walk distance; *V*′_O_2__: oxygen consumption; *V*′_E_/*V*′_CO_2__ slope: slope relating minute ventilation to carbon dioxide production ratio; RER: respiratory exchange ratio; HR: heart rate; *P*_etCO_2__: end-tidal partial pressure of carbon dioxide; CPET: cardiopulmonary exercise testing; RAP: right atrial pressure; PAP: pulmonary arterial pressure; PAWP: pulmonary arterial wedge pressure; PVR: pulmonary vascular resistance.

An adverse event was considered to have occurred if any of the following conditions were met: 1) death; 2) delivery of external defibrillation or implantable cardioverter–defibrillator therapy; 3) sustained ventricular tachycardia; 4) myocardial infarction or urgent referral for coronary angiography and myocardial revascularisation; 5) syncope; 6) use of advanced cardiac life support drugs; or 7) immediate hospital admission [13].

There were no deaths or syncopal episodes. Only two tests (0.05%) met the pre-specified definition of an adverse event. Among the two adverse events, one patient (female, 74 years) with chronic thromboembolic pulmonary hypertension (CTEPH) developed transient anterior ST segment elevation on ECG and mild chest discomfort at peak exercise. She was admitted to the hospital and a coronary angiography later excluded obstructive coronary disease. The second patient (male, 73 years) with CTEPH developed reversible anterior ST elevation during exercise. Subsequent angiography revealed a significant mid-left anterior descending coronary artery lesion successfully treated with stenting. Both patients made full recoveries, and no patient required resuscitation or emergency medications.

The vast majority of tests (n=3811, 98.5%) were terminated by patient-reported fatigue or dyspnoea, while 56 (1.5%) were stopped pre-emptively by the monitoring staff. Premature termination criteria, according to contemporaneous guidelines, included: angina; significant arrhythmias causing symptoms or haemodynamic compromise; fall in systolic blood pressure >20 mmHg from the highest value during the test; hypertensive response defined as >250 mmHg systolic and >120 mmHg diastolic; severe desaturation (peripheral oxygen saturation <80%; further desaturation was permitted in those with known chronic low saturations at the discretion of the supervising doctor); loss of coordination; mental confusion; and dizziness or faintness [1, 2]. Reasons for test termination in our cohort included: dizziness (n=22, 0.57%), oxygen desaturation (n=12, 0.31%), increased ventricular ectopy (n=8, 0.21%), vasovagal episodes (n=6, 0.16%), abnormal blood pressure response (n=6, 0.16%), and transient ST-segment elevation (n=2, 0.05%).

The six vasovagal episodes occurred in younger participants (median age 28 years, five female), at peak exercise (n=1) or early recovery phase (n=5). They were characterised by a drop in blood pressure without loss of consciousness and all recovered promptly without intervention (grade 2 – Common Terminology Criteria for Adverse Events v6, 2025) [14].

Our present series is, to our knowledge, the largest single-centre report of CPET safety in PH to date. The absence of serious events in nearly 4000 studies performed over a decade supports the view that incremental, symptom-limited CPET is safe and feasible in both suspected and confirmed PH. Importantly, 33% of our cohort were aged ≥65 years, highlighting that advanced age alone should not preclude testing when adequate supervision and monitoring are ensured.

The overall adverse event rate of 0.05% in this high-risk population compares favourably with previously published cohorts [4, 13, 15]. Skalski *et al.* [13] reviewed 5060 CPETs across 4250 patients with a wide spectrum of high-risk cardiovascular diseases, including heart failure, hypertrophic cardiomyopathy, aortic stenosis and PH, and reported an overall adverse event rate of 0.16%, with no fatalities. Their population included 194 patients (3.8%) with PH, and there were no adverse events within this subset. Similarly, Sun *et al.* [4] reported no adverse events in 53 patients with primary pulmonary hypertension undergoing CPET, despite severely reduced exercise capacity and haemodynamic compromise. Together, these findings underscore that when appropriately screened and supervised, CPET is feasible and safe, even in advanced cardiopulmonary disease.

Several elements likely contributed to this safety profile. First, all tests were symptom-limited and conducted with strict adherence to Association for Respiratory Technology and Physiology/ATS criteria for termination, and avoiding step protocols that risk excessive cardiovascular strain. Second, all participants were clinically stable at the time of testing, with explicit exclusion of those requiring oxygen or with recent syncope. Third, the cycle ergometer modality minimised falls or trauma risk compared with treadmill testing. Fourth, all procedures were performed in a specialist PH/physiology centre with immediate medical support and experienced staff.

This study has some limitations, mainly related to its retrospective and single-centre design, which limit generalisability. However, our findings are in line with prior literature on the subject, providing convergent evidence that CPET, when performed within recommended safety frameworks, carries negligible risk even in advanced pulmonary vascular disease.

In conclusion, this 10-year experience from a UK national PH centre demonstrates that incremental, symptom-limited CPET is safe in patients with suspected or confirmed PH, with no deaths or syncopal events and an adverse event rate of only 0.05%. When performed under appropriate supervision, CPET should be regarded as a routine and low-risk investigation in PH. Our results reinforce international guideline recommendations and provide the largest dataset to date supporting the safety of CPET in this population.
